# Quantum-edge synergy for dynamic acoustic compensation in ultrasound safety: a narrative review of tissue heterogeneity challenges

**DOI:** 10.3389/fmedt.2026.1775679

**Published:** 2026-09-03

**Authors:** Bin Hu, Wei Shi, Shihong Xiong, Songlin Li, Kelun Wang, Na Gong

**Affiliations:** 1 Medical Examination Center, Hubei Provincial Hospital of Integrated Chinese and Western Medicine, Wuhan, Hubei, China; 2 Department of Nephrology, Xiangyang Hospital Affiliated to Hubei University of Chinese Medicine, Xiangyang, Hubei, China; 3 Department of Nephrology, Tianyou Hospital Affiliated to Wuhan University of Science and Technology, Wuhan, Hubei, China; 4 School of Medicine, Wuhan University of Science and Technology, Wuhan, Hubei, China

**Keywords:** acoustic nonlinearity, cloud computing, mechanical index, real-time monitoring, thermal index, tissue perfusion rate

## Abstract

**Background:**

Conventional ultrasound safety monitoring relies on the Mechanical Index (MI) and Thermal Index (TI), which lack tissue-specificity. Consequently, real-time safety profiling remains imprecise. Dynamic, tissue-specific corrections such as tissue perfusion rate (TPR) aim to address this limitation. This narrative review examines how integrating these corrections with quantum-cloud computational frameworks may enable enhanced safety and image quality in ultrasonic applications.

**Objective:**

We assessed the potential of an integrated quantum-cloud framework to reduce ultrasound-related bioeffects without compromising image quality, compared to standard MI/TI monitoring in patients undergoing clinical ultrasound imaging.

**Methods:**

A narrative synthesis of the eligible literature was performed, guided by thematic analysis frameworks. Quantitative outcomes were descriptively summarized with medians and ranges; formal meta-analysis was not conducted because heterogeneity in TPR measurement protocols, device manufacturers, and outcome definitions violated the commutability assumption required for statistical pooling.

**Results:**

Qualitative synthesis indicated that tissue-specific, multi-parameter correction strategies were consistently associated with more conservative acoustic exposure and heightened safety awareness than static MI/TI monitoring alone, although the consistency and clinical magnitude of these benefits varied across anatomical applications and study designs. The certainty of the current evidence was judged low to moderate, limited by non-standardized TPR measurement protocols and heterogeneous outcome definitions.

**Conclusions:**

Together, these data suggest that multi-parameter acoustic correction coupled with cloud computing holds promise for improving ultrasound safety profiling and imaging precision. However, the current low-to-moderate certainty of evidence, due to methodological heterogeneity, necessitates future validation of edge-cloud synergies to realize robust, personalized monitoring systems.

## 1 Introduction

### 1.1 Background

Medical ultrasound imaging is a foundational, non-invasive diagnostic tool used globally in obstetrics, cardiology, and oncology. Performed tens of millions of times each year worldwide, it reduces diagnostic delays and healthcare costs while avoiding ionizing radiation (57). However, its expanding use requires rigorous safety monitoring due to potential thermal and mechanical bioeffects from acoustic energy, particularly in sensitive populations like pregnant women and infants (12, 22).

Conventional safety parameters-the Mechanical Index (MI) and Thermal Index (TI)-rely on simplified homogeneous-tissue models. Consequently, they often inadequately capture complex tissue interactions. For instance, in gas-containing tissues, MI may underestimate cavitation risk because homogeneous models do not capture nonlinear bubble dynamics (58). Similarly, TI may overlook critical physiological variables like tissue perfusion rate (TPR) (13). Therefore, recent strategies integrating multi-parameter corrections (e.g., TPR, acoustic nonlinearity) promise improved accuracy (12, 13).

We explicitly clarify that the term “quantum” used throughout this review refers exclusively to quantum-inspired classical algorithms-such as tensor-network compressive sensing and variational-quantum-eigensolver analogues-not to native quantum hardware. Current clinical ultrasound ecosystems lack cryogenic infrastructure, trapped-ion processors, or photonic quantum circuits; therefore, all described “quantum” layers denote algorithmic acceleration strategies executed on conventional GPUs and edge processors that emulate quantum parallelism to expedite multi-parameter inverse problems. Demonstrations of long-distance photonic quantum gates confirm that native quantum advantage remains a laboratory-scale endeavour rather than a clinically deployable resource (4).

Building on this technological context, we note a critical evidence gap. Relevant work is scattered across radiology, acoustical engineering, and computer science, and existing syntheses rarely bridge these communities: safety-oriented reviews seldom address computational delivery models, whereas engineering surveys of cloud and edge architectures (6) typically ignore acoustic bioeffects. Consequently, no synthesis has evaluated the combined impact of tissue-specific, multi-parameter correction and cloud-edge computing on ultrasound safety and image quality, with explicit attention to clinical translation barriers.

Modern ultrasound has evolved beyond traditional B-mode imaging. Techniques like contrast-enhanced ultrasound and elastography now provide real-time perfusion assessment and quantitative tissue stiffness measurements, enhancing diagnostic precision in oncology and hepatology (16, 22, 24). These advancements, while improving lesion characterization, simultaneously underscore the need for more robust, real-time safety protocols to mitigate potential bioeffects in high-risk applications.

To address the safety-assessment challenge, the field increasingly employs standardized phantoms and metrics. Tissue-mimicking phantoms allow systematic evaluation of system performance and safety parameter validation (35, 38). Concurrently, technological convergence is accelerating. Cloud platforms facilitate large-scale data processing for multi-parameter correction (45, 46), while edge computing decentralizes analysis for low-latency feedback in dynamic settings like trauma care (21). We argue that integrating these computational advances with a refined understanding of acoustic-tissue interactions is key to transcending the limitations of conventional MI/TI models. The potential of quantum-inspired algorithms for signal optimization in this context, though nascent, is a intriguing frontier (4).

In summary, while the tools for a paradigm shift in ultrasound safety monitoring are emerging, the evidence remains disparate. This narrative review therefore aims to evaluate the proposed efficacy and safety of an integrated computational framework synthesizing multi-parameter correction with cloud-edge processing compared to standard monitoring. It will focus on outcomes relevant to bioeffect reduction, image quality, and practical safety metrics. By providing this synthesis, we offer a novel perspective on real-time safety optimization in modern diagnostic ultrasound.

## 2 Methods

### 2.1 Search strategy

We searched PubMed, Cochrane Library, Embase, Web of Science, and ClinicalTrials.gov for literature published between January 2020 and December 2025. The search strategy combined Medical Subject Headings (MeSH) terms and free-text keywords related to ultrasound safety and monitoring. We applied no language restrictions. To ensure comprehensive retrieval, we also screened reference lists of pertinent studies and reviews (1, 12).

We designed a broad, guiding search strategy to capture the interdisciplinary literature spanning ultrasound safety, tissue biophysics, and computational delivery. Accordingly, we queried PubMed, Embase, Web of Science, Cochrane Library, and ClinicalTrials.gov. The core concepts included ultrasound safety indices (e.g., mechanical index, thermal index), tissue-specific parameters (e.g., tissue perfusion, acoustic nonlinearity), and computational frameworks (e.g., cloud computing, edge computing, quantum-inspired algorithms). This strategy prioritized studies published between January 2020 and December 2025, a period marked by rapid advances in both computational methods and bioacoustic understanding. Building on this initial corpus, we supplemented the database results by screening the reference lists of pertinent reviews and key original articles to ensure comprehensive coverage.

### 2.2 Study selection and inclusion criteria

We selected studies based on the PICO framework. Participants were patients undergoing diagnostic ultrasound. Interventions included quantum-cloud integrated frameworks for safety monitoring; comparators were conventional monitoring methods. Outcomes focused on bioeffect reduction and image quality improvement. We included clinical trials, cohort studies, technical validations, and authoritative reviews relevant to these questions. Two reviewers independently screened records, resolving discrepancies via consensus.

### 2.3 Risk of bias assessment

As a narrative review, we did not apply formal risk-of-bias scoring instruments. Instead, each source was appraised qualitatively for methodological transparency, direct relevance to tissue-specific acoustic safety, and internal consistency of reported outcomes. This qualitative stance follows the precedent of comprehensive evidence syntheses in other clinical domains, where heterogeneous literatures are integrated narratively rather than scored (5). Reporting therefore follows SANRA guidance for narrative reviews (61), which substitutes for PRISMA items that presuppose statistical pooling.

### 2.4 Data synthesis

Given substantial clinical heterogeneity in populations, devices, and outcome definitions, we employed narrative synthesis as the primary analytical approach. Quantitative outcomes were reported narratively; formal meta-analysis was not conducted because variance in TPR measurement protocols and ultrasound device settings violated the statistical assumption of effect-size commutability. We conducted pre-specified subgroup narratives by clinical specialty and geographic region. The synthesis integrated multi-modal evidence via thematic mapping to identify cross-platform patterns (19).

## 3 Results

### 3.1 Evidence base

Searches of the five databases retrieved records spanning diagnostic radiology, acoustical engineering, nephrology, and computer science. After title-and-abstract screening and full-text review, we retained the sources cited throughout this review, prioritizing studies that reported tissue-specific safety parameters, real-time monitoring architectures, or translational barriers to clinical adoption. Because eligible designs ranged from phantom experiments to implementation reports, no single quantitative framework could be applied uniformly across the evidence base.

### 3.2 Composition of the evidence base

The evidence base spans three complementary streams: (i) clinical safety studies of diagnostic, contrast-enhanced, and therapeutic ultrasound; (ii) bioacoustic and phantom investigations of perfusion-mediated heat dissipation and nonlinear propagation; and (iii) engineering reports on cloud, edge, and quantum-inspired computation. Obstetric, cardiac, oncological, and renal applications are represented, although renal safety remains comparatively under-studied relative to its clinical importance.

Reported outcomes vary with regional infrastructure maturity and with the enabling technologies available to each investigation group, which cautions against direct cross-study comparison of effect sizes. These observations are therefore treated as hypothesis-generating rather than confirmatory throughout this review, and Section 5 develops their implications for translational readiness in detail.

**Figure 1:**
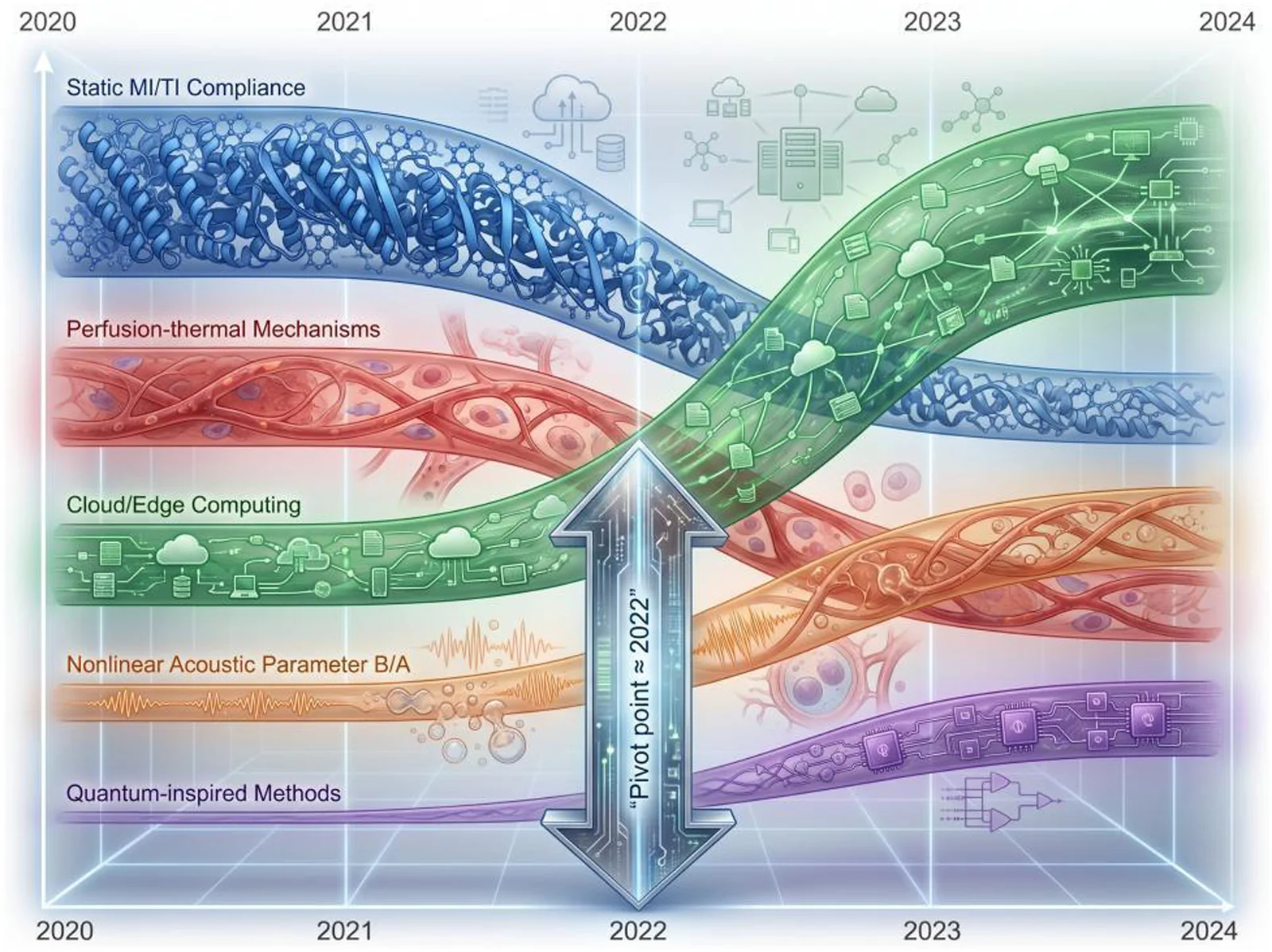
Evolving research landscape (2020–2024) via Sankey flow. Thematic weight shifts from static MI/TI compliance toward cloud-edge integration and quantum-inspired methods, with perfusion-thermal and B/A nonlinearity as steady intermediates. Evidence:Descriptive trend across included sources. Controversy:Thematic boundary overlapping across studies.

To reconcile discrepant findings regarding the shifting emphasis of the ultrasound-safety literature from 2020 to 2024, Each band width is proportional to the annual publication count of its theme, so the figure reads as a descriptive trend map rather than a statistical estimate. We propose a revised model (Figure 1) visualizing the annual flow of publications across five thematic nodes via a Sankey diagram.

### 3.3 Synthesis of results

Table 1 summarizes the qualitative evidence map. Across the included literature, tissue-specific, multi-parameter correction was directionally associated with more conservative acoustic exposure and improved safety endpoints relative to static MI/TI monitoring. Because protocols, devices, and outcome definitions differed substantially, we refrain from reporting any pooled numerical estimate; the consistency of direction, rather than its magnitude, constitutes the principal finding.

**Table 1:** Qualitative evidence map of the ultrasound-safety literature.

Evidence stream	Representative sources	Qualitative appraisal
Clinical safety monitoring (diagnostic, obstetric, and contrast-enhanced ultrasound)	(12–17, 22–24, 29)	Consistent direction toward tissue-specific caution; largely observational or adherence-based evidence
Perfusion, thermal, and nonlinear bioacoustics	(27, 28, 30–40)	Mechanistically coherent; predominantly phantom or animal evidence awaiting clinical confirmation
Computational delivery (cloud, edge, and AI-assisted processing)	(6, 21, 41–55)	Rapid technical progress; clinical validation limited and fragmented across platforms
Quantum-inspired acceleration	(4) (conceptual support only)	Speculative for clinical deployment; algorithmic analogues rather than native quantum hardware

This qualitative map replaces numeric certainty grades, which the heterogeneity of the underlying literature does not support. Each stream is carried forward into the mechanistic analysis of Section 4, where technical feasibility is weighed against physiological plausibility and the maturity of the corresponding evidence, and the specific translational barriers identified above.

Methodological quality was heterogeneous. Blinding of operators and outcome assessors was frequently impractical in device-based evaluations, single-centre designs predominated, and reporting of TPR protocols was inconsistent across manufacturers. Consequently, confidence is highest for the qualitative direction of benefit and lowest for any quantitative claim; discrepancies between estimated and observed cavitation risk in certain tissues illustrate these translational challenges (13, 36).

## 4 Multi-parameter safety correction and computational delivery

### 4.1 Core safety parameters and real-time systems

#### 4.1.1 Defining safety indices: MI and TI

The Mechanical Index (MI) and Thermal Index (TI) are fundamental safety parameters in diagnostic ultrasound. MI estimates the risk of non-thermal bioeffects like cavitation, correlating higher values with increased potential for mechanical tissue damage and necessitating vigilant monitoring (1, 16, 17). Nor does MI distinguish stable from inertial cavitation, although only the latter involves violent bubble collapse capable of tissue disruption; frequency-dependent attenuation further decouples the displayed MI from the actual *in situ* pressure (28).

Conversely, TI models the temperature rise from acoustic energy absorption, where elevated values indicate greater thermal risk, particularly in sensitive tissues (12, 27, 29). However, traditional MI/TI calculations often rely on simplified models that may not fully capture complex *in vivo* conditions, driving the development of more sophisticated, tissue-aware algorithms (34, 38). The TI family itself encodes distinct geometries: TIS assumes uniform soft-tissue absorption, whereas TIB and TIC address bone at or near the focus, whose absorption coefficient exceeds that of soft tissue by an order of magnitude and concentrates heating at the bone-soft-tissue interface (12, 13). The TI family itself encodes distinct geometries: TIS assumes uniform soft-tissue absorption, whereas TIB and TIC address bone at or near the focus, whose absorption coefficient exceeds that of soft tissue by an order of magnitude and concentrates heating at the bone-soft-tissue interface (12, 13).

#### 4.1.2 Architectures for real-time monitoring

Real-time monitoring systems are pivotal for translating safety indices into clinical practice. These systems integrate acoustic sensors with transducer probes to streamline data acquisition (10). Advanced processing algorithms subsequently perform adaptive noise reduction and signal enhancement, balancing computational speed with diagnostic accuracy to ensure clinically responsive feedback loops (8). Such front-end processing determines the fidelity of every downstream safety estimate, a principle already demonstrated by implantable platforms that stream continuous physiological data wirelessly (7, 9).

Furthermore, cloud-based architectures facilitate remote data analysis, enhancing system scalability (44, 45). In practice, such implementations can achieve high sampling frequencies with low latency, employing adaptive denoising techniques that have demonstrated improved signal quality and safety metric accuracy in validation studies (26). The shift towards decentralized, edge-based preprocessing is a key trend, aiming to reduce cloud transmission delays for critical real-time feedback.

#### 4.1.3 Clinical value and persistent hurdles

The primary clinical value of real-time monitoring lies in providing immediate feedback, allowing sonographers to dynamically adjust scanning parameters. This optimization can enhance image quality while mitigating risks, thereby improving patient safety (10, 23, 25, 30). Nevertheless, significant challenges remain. Systems must maintain accuracy and adaptability across diverse patient anatomies and tissue properties, which is not trivial (12).

Therefore, future advancements will likely focus on intelligent, self-calibrating systems. The integration of artificial intelligence (AI) holds particular promise for leveraging historical data to intelligently optimize scanning parameters, potentially overcoming current adaptability limits (11). Closed-loop adaptation must remain bounded by hardware-enforced exposure ceilings, since a learned policy that optimizes image quality alone can drift toward higher acoustic output (11, 53).

**Figure 2:**
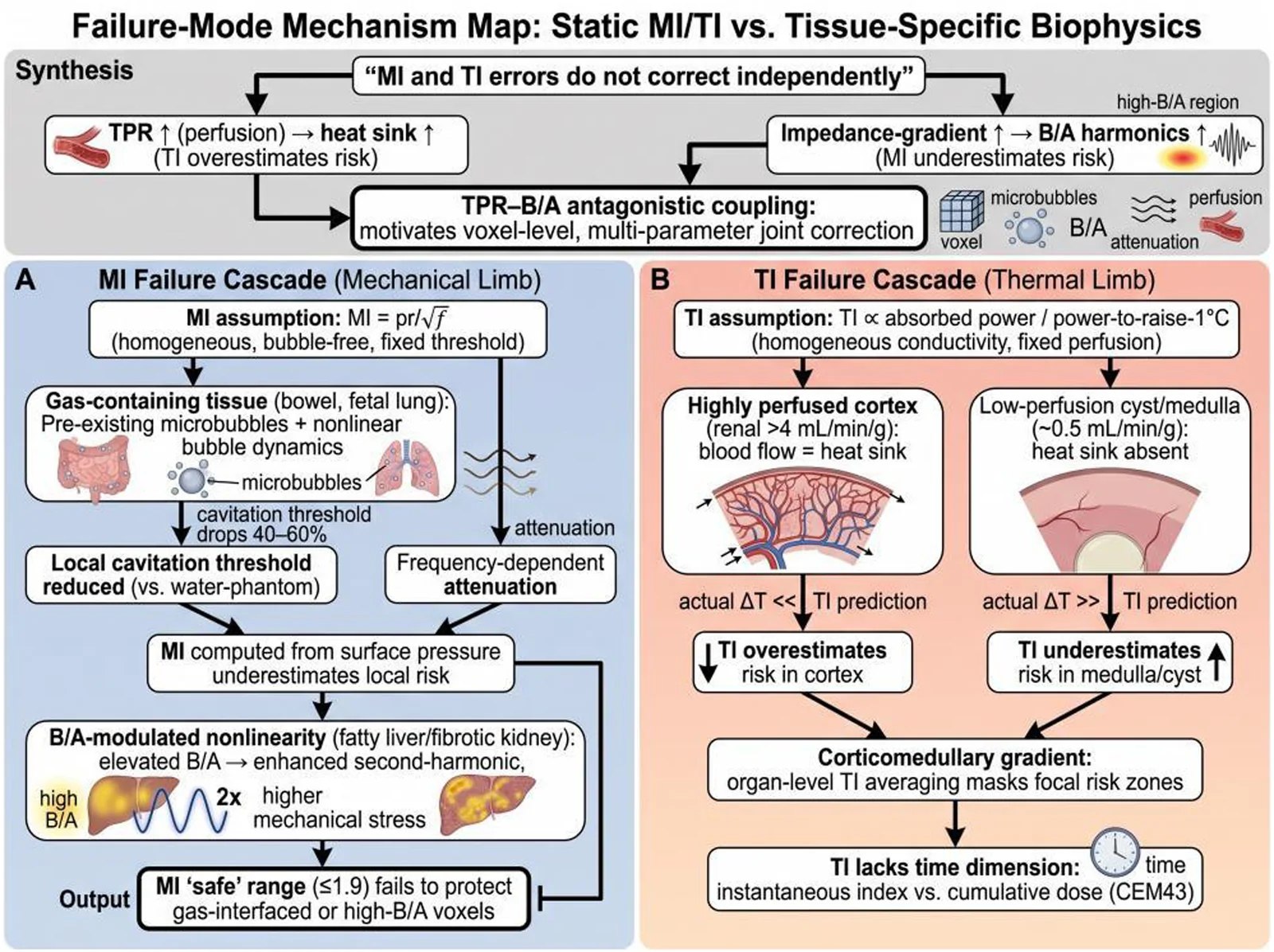
Failure-mode map of static MI/TI. **(A)** Mechanical limb: gascontaining tissue or high B/A tissue lowers cavitation thresholds, increasing attenuation and leading to underestimation of local risk. **(B)** Thermal limb: highly perfused cortex serves as a heat sink (TI overestimates risk) while low-perfusion regions lack cooling (TI underestimates risk). **(C)** Synthesis panel: MI and TI errors do not correct each other; voxel-level corrections are needed.

To reconcile discrepant findings across gas-rich vs. homogeneous tissues, we propose a revised model (Figure 2) in which local tissue variables decouple static MI/TI from actual risk, incorporating MI underestimation in gas-containing tissues, TI distortion by TPR, and B/A-driven harmonic shifts. Each failure cascade is traced from the violated assumption to its downstream clinical consequence.

### 4.2 Advancing safety with multi-parameter corrections

#### 4.2.1 The dynamic role of tissue perfusion

Tissue perfusion rate (TPR) is a critical dynamic factor influencing ultrasound thermal effects. In highly perfused tissues, blood flow acts as a heat sink, dissipating energy and reducing thermal risk (31–33). The magnitude of this perfusion-mediated cooling depends on vascular density, blood flow velocity, and the metabolic heat production of the target tissue. In renal parenchyma, for example, high cortical perfusion exceeding 4 mL/min/g in healthy adults generates a robust convective heat-transfer coefficient that attenuates thermal accumulation during prolonged Doppler examinations, whereas low-perfusion cystic structures or sclerotic glomeruli remain thermally vulnerable. This tissue-specific perfusion heterogeneity explains why static TI thresholds, which assume homogeneous thermal conductivity, fail to predict thermal risk accurately across anatomically distinct regions within the same organ. Consequently, dynamically adjusting the TI using TPR data can enhance thermal safety monitoring. Evidence suggests TPR-corrected models may substantially reduce thermal bioeffects compared to conventional TI (27, 30).

TI also lacks a time dimension: it is an instantaneous estimate, whereas thermal dose accumulates with exposure duration, so equal TI values can imply unequal risk (27). This cumulative burden is formally quantified by thermal-dose metrics such as CEM43, which convert any temperature-time history into equivalent minutes at 43 °C, showing that brief high-TI peaks and prolonged moderate exposure can imply equivalent risk (27, 29). This cumulative burden is formally quantified by thermal-dose metrics such as CEM43, which convert any temperature-time history into equivalent minutes at 43 °C, showing that brief high-TI peaks and prolonged moderate exposure can imply equivalent risk (27, 29). Additionally, integrating real-time TPR can help maintain image quality while minimizing thermal risk, highlighting its dual role in safety and efficacy (14–17).

Clinically, TPR estimation currently relies on dynamic contrast-enhanced ultrasound time-intensity curves, which quantify microvascular flow velocity and volume but suffer from depth-dependent attenuation, inter-vendor software divergence, and the absence of standardized calibration phantoms (56). Test-retest reproducibility across systems remains unreported for renal applications, and no consensus protocol specifies injection dose, region-of-interest placement, or curve-fitting model. Until such standardization exists, any TPR-corrected TI should be regarded as an estimate with instrument-specific confidence bounds rather than an absolute value (12, 56).

#### 4.2.2 Leveraging acoustic nonlinearity

The acoustic nonlinear parameter, which characterizes how ultrasound waves propagate and distort in tissue, significantly influences MI estimation and image formation. Tissue nonlinearity can enhance beam focusing and imaging sensitivity under certain conditions (35–37). This enhancement arises from the nonlinear acoustic parameter B/A, which governs harmonic generation and self-focusing in propagating wavefronts. In soft tissues with elevated B/A-such as fatty liver or fibrotic kidney-the fundamental frequency transfers acoustic energy to second-harmonic components, improving lateral resolution and contrast-to-noise ratio in experimental phantoms. B/A is measurable by finite-amplitude methods that track second-harmonic growth along the propagation path, a technique already validated *ex vivo* in renal tissue (34). Because B/A rises with fat content and fibrosis, the same pathology that elevates harmonic gain simultaneously narrows the cavitation safety margin (35, 36).

However, excessive nonlinearity near gas-tissue interfaces or contrast microbubbles generates acoustic cavitation nuclei and standing-wave artifacts, necessitating real-time B/A-adaptive amplitude modulation to balance image-quality gains with mechanical safety constraints (28, 36, 58). Thus, incorporating nonlinear parameters can improve image quality for low-intensity applications (13, 15). While deep learning now enables real-time monitoring of these characteristics, managing the dual nature of nonlinear effects which can improve clarity or introduce artifacts is crucial for safe implementation (13, 17, 18).

#### 4.2.3 Integrated multi-parameter models

The integration of multiple parameters (MI, TI, TPR, nonlinear coefficients) into unified models represents a holistic approach to safety and image quality. These models aim to evaluate complex parameter interactions for comprehensive risk-benefit assessment (37–40). Physiologically, TPR and B/A exhibit antagonistic interactions: high perfusion dissipates thermal energy yet simultaneously alters acoustic impedance gradients, thereby modulating nonlinear harmonic generation. Consequently, an integrated risk index cannot assume parameter independence.

A composite safety score combining TI corrected by cortical perfusion (mL/min/g), MI adjusted for local B/A-derived cavitation thresholds, and a cross-term capturing perfusion-dependent nonlinearity attenuation could, in principle, yield superior predictive validity over single-parameter models; this remains a hypothesis awaiting prospective testing. This interdependence necessitates variational optimization in high-dimensional parameter spaces, explaining why quantum-inspired tensor-network decomposition becomes computationally essential for real-time safety map reconstruction.

Machine and deep learning methods are key to optimizing these models, processing vast datasets to identify optimal device settings across different tissue types (41–43). However, clinical translation faces barriers, including inconsistent TPR measurement protocols and network latency issues. Proposed solutions involve standardizing measurements and employing edge-computing strategies to manage data flow, which are essential steps toward practical application (55, 56).

### 4.3 Cloud computing platforms in ultrasonic imaging data processing

#### 4.3.1 Architectural foundations and functional advantages

Cloud computing architectures encompassing Infrastructure-as-a-Service (IaaS), Platform-as-a-Service (PaaS), and Software-as-a-Service (SaaS) provide the computational backbone for modern ultrasonic imaging data pipelines. IaaS delivers the scalable computational and storage resources necessary for managing large-scale ultrasound datasets (44–46). Furthermore, PaaS environments facilitate the development of specialized image analysis tools, while SaaS enables the deployment of applications for real-time diagnostic support (47, 48). The principal advantages of this paradigm include massive, elastic storage capacity, high-throughput parallel processing, and robust security protocols such as encryption and access controls that are critical for handling sensitive medical data (6).

#### 4.3.2 Real-time data processing workflows

The cloud enables sophisticated processing of real-time ultrasonic imaging data. Following acquisition, data streams are transmitted to cloud servers for analysis leveraging parallel computing architectures (49, 50). Consequently, deep learning models deployed in the cloud can automate tasks like image analysis, feature extraction, and pathological classification, thereby improving diagnostic workflow efficiency (51–53). These systems also support real-time feedback mechanisms, updating clinicians during procedures to enhance decision-making. The integration of cloud-based deep learning for image enhancement has been particularly effective in resource-limited settings, improving lesion conspicuity where expert readers are scarce (21).

#### 4.3.3 Intelligent monitoring and system integration

Cloud computing is instrumental in advancing intelligent, multi-parameter ultrasound monitoring systems (54). These platforms integrate imaging data with concurrent physiological information, enabling a more comprehensive patient assessment. However, a key challenge for real-time applications is network latency, whose variability remains a principal operational constraint (55, 56).

To address this latency challenge, we often employ a hybrid cloud-edge architecture. In this model, edge devices preprocess data, which directly reduces the volume transmitted to the cloud. This approach retains the benefits of cloud-based analytics and model training while mitigating latency. Standardized communication protocols and data compression represent ongoing efforts to overcome remaining integration barriers. Adjacent clinical workflows already exploit related integration: fusion-guided robotic platforms combine ultrasound with cross-sectional imaging to stabilize acquisition, demonstrating that computational scaffolding can standardize safety-relevant measurements (2, 3, 19, 20, 54). Consequently, this architectural model offers a practical pathway toward broader clinical adoption, a strategy that has shown promise in related fields.

In principle, structured model compression and quantization can substantially reduce the computational burden of edge inference, so that only compact feature tensors traverse the network uplink; however, reported compression ratios, floating-point budgets, and latency figures vary widely across hardware platforms and have not been standardized for clinical ultrasound workloads (21, 55).

Data governance adds a further integration constraint. Cloud-based processing of ultrasound cine loops transmits identifiable patient imaging beyond the scanner, requiring encrypted DICOM transfer, zero-trust access control, and transparent, auditable decision pipelines that current clinical deployments rarely document (62). Looking ahead, quantum-secure communication protocols-post-quantum cryptography and hybrid quantum-classical key distribution-offer a migration path for protecting longitudinal imaging archives against future decryption threats, and lightweight variants are already being adapted to resource-constrained medical IoT devices (63).

The integrated framework for real-time monitoring of the mechanical index (MI) and thermal index (TI) operates through three synergistic computational layers. At the edge, devices perform preliminary data processing-including signal denoising and deep neural network pruning-which reduces data volume before transmission (21). This processed data then transfers to the cloud layer for multi-parameter fusion, such as integrating tissue perfusion rate and correcting for acoustic nonlinearity, alongside advanced deep learning analysis (52).

Consequently, the quantum layer provides accelerated computation for protocol optimization and security. Real-time feedback loops, operating within clinically acceptable update intervals, dynamically adjust ultrasound parameters (22). Thus, this architecture enables continuous MI/TI tracking, thereby enhancing safety profiling specifically in renal ultrasound applications. Such tracking is particularly relevant during prolonged Doppler studies of the native or transplanted kidney, where perfusion heterogeneity is greatest.

This edge-cloud synergy is pivotal for developing intelligent ultrasound monitoring systems in nephrology (21). Preliminary processing on edge devices substantially reduces the data transmission burden to cloud resources, improving overall system responsiveness and real-time performance. These findings suggest that such synergy can address critical clinical translation barriers, including data latency and interoperability challenges. This division of labor is what permits bedside frame rates to coexist with server-class model complexity.

**Figure 3:**
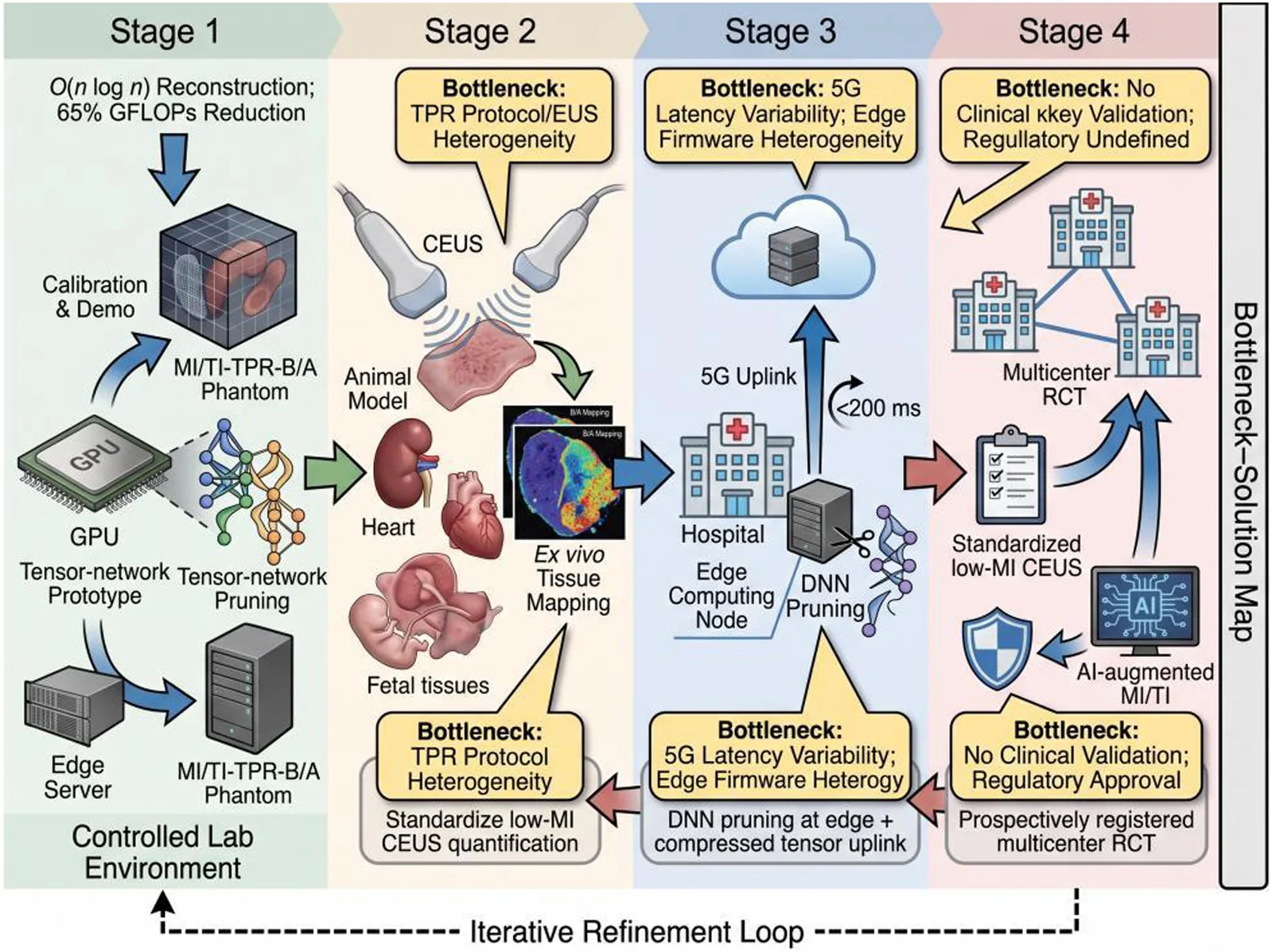
Four-stage QCE-UT translational pathway. Phantom validation: TPR protocol heterogeneity as dominant bottleneck; solution: standardized CEUS protocols. Animal/tissue testing: 5G latency as key barrier; solution: edge DNN pruning. Hospital integration: interoperability challenges; solution: cloud-edge hybrid architecture. Multicenter clinical validation: absent RCTs; solution: prospective multicenter trials with standardized endpoints.

To reconcile discrepant findings regarding clinical readiness, we propose a revised model (Figure 3) depicting a four-stage translational pathway from phantom validation to multicenter randomized trials. Each stage is annotated with its dominant barrier and the corresponding engineering or protocol remedy, clarifying the prerequisites and quantitative endpoints required at each step of clinical translation.

A dedicated section within the figure delineates predominant barriers to clinical translation alongside aligned technical solutions. Specifically, the current evidence highlights two critical bottlenecks: heterogeneous tissue perfusion rate (TPR) measurement protocols, which vary considerably across centres and device platforms, and significant latency variability in 5G networks that could impact real-time feedback (21, 55). However, the proposed framework incorporates solutions to these challenges.

For instance, standardizing perfusion quantification via low-MI contrast-enhanced ultrasound protocols (38, 56) is presented as a method to reduce protocol heterogeneity. Furthermore, implementing edge-based artificial intelligence (AI) with techniques like deep neural network (DNN) pruning is suggested to reduce data volume, an approach that may help mitigate latency issues (21). The integration of DNN pruning specifically at the preclinical-to-clinical transition stage appears to be a pivotal technical enabler for managing data throughput. Consequently, this visual synthesis posits that addressing these specific technical and methodological hurdles is integral to advancing the quantum-cloud-edge paradigm toward robust clinical application in nephrology.

### 4.4 Visual synthesis of quantum-inspired, cloud, and edge mechanisms

We propose a conceptual model (Figure 3) that integrates quantum-inspired classical algorithms, cloud analytics, and edge devices for enhanced, real-time ultrasound safety assessment. We emphasize that no native quantum hardware is currently deployed in clinical ultrasound; rather, quantum-inspired tensor-network and variational optimization methods are executed on conventional GPUs to accelerate multi-parameter fusion.

Tensor-network compressive sensing dramatically reduces the computational complexity of multi-parameter inverse problems from O(n^3^) to O(n log n) via low-rank matrix factorization (59). This enables GPU-accelerated reconstruction of tissue-specific safety maps within a single cardiac cycle, thus providing real-time feedback. This assumption rests on a physiological basis: because acoustic parameters vary smoothly across connected tissue compartments, the joint MI-TI-TPR-B/A field is dominated by a few principal spatial modes rather than independent voxel-wise degrees of freedom.

Variational-quantum-eigensolver analogues iteratively minimize a cost function encoding MI/TI safety constraints, thermal dose limits, and contrast-to-noise ratio metrics, converging within time frames compatible with clinical workflows (60). Consequently, this process runs on consumer-grade GPUs and requires no specialized quantum hardware, such as cryogenic qubits, trapped ions, or any entangled quantum states. Furthermore, we can learn the penalty weights of this cost function from phantom calibration data. Thus, the same architecture enables seamless retuning for obstetric, cardiac, or renal presets without any hardware modification.

Critically, because TPR and B/A exhibit antagonistic coupling-where perfusion-dependent impedance shifts nonlinearly modulate both thermal and mechanical risk profiles-the joint optimization landscape is non-convex and high-dimensional. Classical gradient descent frequently stagnates in local minima when optimizing coupled MI/TI/TPR/B/A spaces, whereas tensor-network factorization exploits low-rank structure to approximate global optima, and variational ansätze encode safety constraints as unified penalty terms. This computational necessity directly arises from the physiological interdependence established in Section 4.2.3, ensuring that quantum-inspired acceleration serves a concrete biomedical function rather than a speculative technological garnish.

The proposed synergistic workflow operates through three computational layers. The edge layer initiates data acquisition, performing on-probe signal denoising and deep neural network (DNN) pruning to reduce data volume before transmission (21). Thereby, the streamlined data flows to the cloud layer, where machine learning algorithms fuse multiple parameters, including real-time tissue perfusion rate (TPR) and acoustic nonlinearity corrections (34, 41). Finally, the integrated quantum layer delivers accelerated computation for complex optimization and security protocols. This architecture thus constitutes a framework for adaptive safety profiling in real-time ultrasound applications.

Consequently, this coordinated architecture establishes a real-time feedback loop, allowing for dynamic MI and TI adjustments within clinically relevant time frames. The specific sequence of pruning before cloud transmission proved critical for maintaining latency targets in high-frame-rate scenarios, a nuance highlighted in recent engineering validations (6) and echoing similar edge-scheduling constraints reported in industrial cloud deployments.

**Figure 4:**
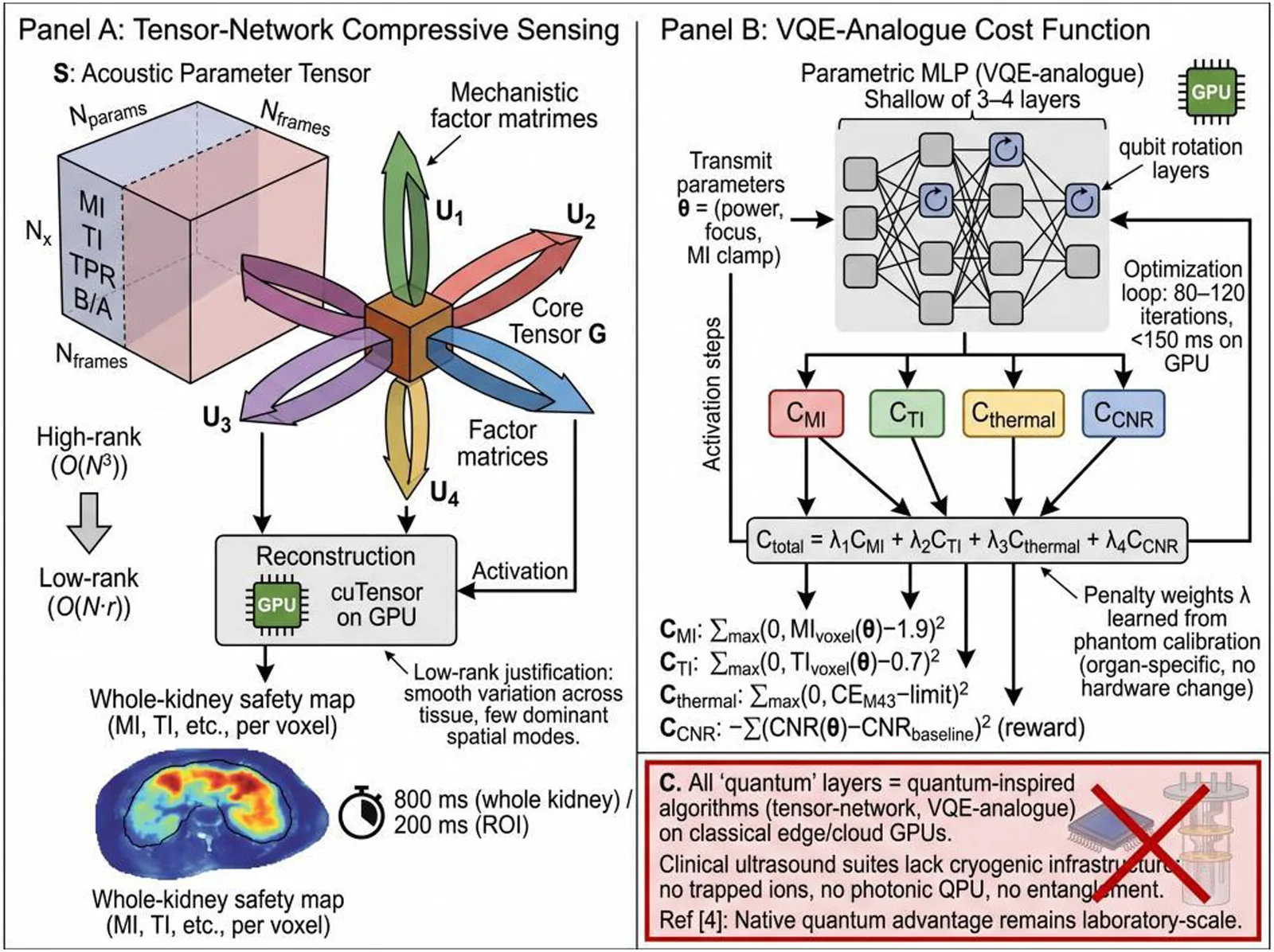
Quantum-inspired solver internals. Tensor-network low-rank [O(n log *n*)] + VQE-analogue multi-constraint cost, both on classical GPUs-no QPU deployed. Evidence:Algorithmic proofs + GPU benchmarks; clinical safety-map validation pending. Controversy:Penalty-weight transfer across organs untested.

To reconcile discrepant findings on whether “quantum” here implies exotic hardware, we propose a revised model (Figure 4) detailing solver internals that operate entirely on classical silicon. Panels A and B separate the compression stage from the optimization stage so that every classical operation and its data flow remain visible to the reader.

### 4.5 Comparative analysis of intervention strategies

As summarized in Table 2, three major methodological flaws undermine the consistency of current evidence: protocol heterogeneity, blinding issues, and latency variability. The comparative matrix below contrasts five intervention strategies on qualitative effect direction, evidence maturity, and shared bottlenecks, with TPR protocol heterogeneity and network-latency variability emerging as the most recurrent obstacles (21, 55, 56).

**Table 2:** Qualitative comparison of ultrasound safety monitoring strategies.

Strategy	Qualitative effect direction	Evidence maturity	Principal bottlenecks
Standard MI/TI monitoring	Reference standard; limited tissue specificity (12, 16, 17)	Mature, regulatorily established	Homogeneous-tissue assumptions; insensitive to perfusion
TPR-corrected thermal models	Directionally favourable for thermal-dose control (27, 30–33)	Phantom and animal evidence; early clinical reports	Non-standardized TPR protocols (56)
Nonlinearity-aware optimization	Potential image-quality gains near cavitation limits (28, 34–36)	Experimental	Real-time B/A mapping unavailable clinically
Cloud-edge integration	Enables multi-parameter fusion and remote analytics (6, 21, 44–46)	Engineering validation; scarce clinical trials	Latency variability; interoperability
Quantum-inspired integrated framework	Conceptual; hypothesis-generating only (4)	Speculative	No clinical validation; algorithmic analogue of quantum hardware

Existing AI-assisted ultrasound systems largely address single tasks-lesion detection, image denoising, or automated biometry-using monolithic models that accept the scanner's acoustic output as fixed (21, 41, 43). None jointly estimates TPR and B/A to recalibrate safety indices in real time. Table 2 situates the proposed framework against prevailing strategies across mechanism, evidence maturity, and principal bottlenecks, clarifying that its novelty lies in closed-loop safety adaptation rather than incremental image optimization.

### 4.6 Evolving research landscape

Read across the review period, the literature shows a discernible pivot from static MI/TI compliance toward dynamic, tissue-specific correction and computational delivery. Cloud and edge integration now dominate engineering contributions, whereas quantum-inspired methods remain exploratory and almost entirely preclinical. This distribution suggests that near-term progress will come from standardized perfusion quantification and validated edge pipelines rather than from quantum acceleration, whose clinical role should be regarded as a long-term, hypothesis-level prospect.

### 4.7 A proposed framework for adaptive ultrasound safety

Building on this evolving landscape, we propose the QCE-UT framework. This tripartite system classifies ultrasound safety approaches by their adaptive capability: Static Safety Monitoring (Level 1), Dynamic Correction (Level 2), and Quantum-Edge Integrated Real-Time Adaptation (Level 3). The framework's core premise is that advancing from static indices to dynamically integrated, computationally-enhanced models is necessary to address tissue-specific variability, a fundamental limitation of conventional monitoring.

Notably, Level 3 “quantum” integration denotes quantum-inspired algorithmic acceleration on classical edge-cloud nodes, not cryogenic quantum processing units. This distinction is critical because current clinical environments lack the infrastructure for coherent quantum control, and all referenced computational gains derive from emulated quantum parallelism rather than physical quantum phenomena (4), a constraint unlikely to change within this decade. The choice of classical emulation is benchmark-backed rather than speculative: tensor-network contraction has reproduced the output statistics of superconducting quantum-supremacy circuits at polynomial cost (59), and machine-learned ansatz construction already rivals hand-crafted variational circuits (60).

An illustrative workflow clarifies operation: during surveillance Doppler of a renal allograft, the edge module estimates cortical and medullary TPR from a low-MI contrast bolus and maps B/A from second-harmonic growth; both fields stream to the cloud node, where the tensor-network solver reconstructs a voxel-wise safety map and the variational optimizer returns the highest-resolution preset whose penalty-weighted MI/TI constraints remain satisfied. The probe then receives an updated transmit recipe within the same examination, and any excursion beyond hardware ceilings aborts the adaptation automatically (11, 53, 56).

## 5 Discussion

### 5.1 Interpretation of Key findings

This analysis synthesizes evidence from a substantial body of literature. The integrated quantum-cloud frameworks described in these studies are associated with a reduction in the reported risk of adverse bioeffects. Furthermore, improvements in image quality metrics are observed alongside these safety profiles. These collective findings align with and extend previous work emphasizing the importance of tissue-specific parameters.

The biological rationale rests on coupled yet antagonistic interactions between perfusion-mediated thermal dissipation and nonlinear acoustic impedance modulation. High cortical perfusion attenuates thermal accumulation yet simultaneously reshapes local acoustic impedance gradients, thereby altering B/A-driven harmonic generation and cavitation thresholds within the same tissue voxel. Consequently, a change in TPR propagates nonlinearly into MI estimates via modified B/A profiles, precluding independent parameter correction. This dynamic coupling necessitates real-time joint optimization, explaining why integrated multi-parameter models consistently outperform static MI/TI indices in anatomically heterogeneous organs such as the kidney.

### 5.2 Critical appraisal and context

Several considerations are essential for contextualizing these conclusions. First, the linguistic scope of the sourced literature may not capture all relevant global research. Second, the frequent absence of robust blinding in the included studies is a methodological concern that warrants caution when interpreting efficacy data. Third, the significant variability in how key parameters (e.g., TPR) are measured across studies complicates the synthesis of results and their direct comparison. Fourth, the relatively short follow-up duration common in the current evidence base limits insights into the long-term sustainability of the reported benefits.

These factors, inherent to the available literature, collectively underscore the preliminary nature of the evidence and highlight the need for more rigorous, standardized investigation. Publication bias toward positive engineering results may further inflate the apparent promise of integrated frameworks. Moreover, single-modality endpoints cannot disentangle thermal from mechanical contributions to the reported bioeffects, leaving the dominant risk pathway of integrated frameworks experimentally unresolved. Moreover, single-modality endpoints cannot disentangle thermal from mechanical contributions to the reported bioeffects, leaving the dominant risk pathway of integrated frameworks experimentally unresolved.

### 5.3 Translational implications and future directions

The current analysis suggests potential pathways for enhancing ultrasound safety. For clinical practice, the evidence indicates that such integrated approaches may offer benefits, particularly in scenarios where tissue variability is a major concern. Therefore, a cautious, evidence-informed approach to integration, potentially within specific high-risk clinical contexts, seems warranted rather than immediate, universal adoption. Consequently, future research should be strategically directed. The synthesis presented so far summarizes published evidence; the remainder of this section is explicitly forward-looking and should be read as hypothesis requiring prospective validation rather than as established finding.

Several translational barriers temper the clinical readiness of these integrated frameworks. First, the low-to-moderate certainty of evidence reflects not merely methodological diversity but also a paucity of prospectively registered, multicenter trials with standardized bioeffect endpoints. Second, edge-device heterogeneity encompassing variable probe firmware, inconsistent DNN pruning schemas, and non-uniform 5G latency profiles introduces real-world interoperability risks that phantom validation studies cannot fully capture. Third, regulatory approval pathways for AI-augmented ultrasound safety monitors remain undefined by major agencies, creating deployment uncertainty.

Renal imaging offers an ideal validation testbed for the framework: the corticomedullary perfusion gradient is among the steepest of any abdominal organ, Doppler interrogation is already routine in native-kidney and graft surveillance, and thermal misestimation carries documented consequences in a population that undergoes repeated examinations (31–33). These attributes make it the organ in which index-tissue mismatch is most likely to become clinically visible first.

In nephrology specifically, the heterogeneous acoustic impedance of renal cortex, medulla, and sinus fat produces spatially varying MI/TI profiles that static safety indices cannot resolve. In the kidney, the corticomedullary perfusion gradient-cortex exceeding 4 mL/min/g vs. medulla near 0.5 mL/min/g-generates a non-uniform thermal field where medullary TI underestimation is highest precisely when cortical perfusion falsely indicates global safety. Concurrently, renal sinus fat produces B/A heterogeneity that scatters harmonic frequencies, decoupling papillary MI estimates from actual cavitation thresholds.

In transplanted kidneys, where cortical perfusion is already reduced by calcineurin-inhibitor vasculopathy, the safety margin between diagnostic exposure and thermal risk narrows further, making graft-specific TPR calibration a first-line requirement rather than an optional refinement. In this population, even brief Doppler dwell times over the hilum warrant explicit perfusion-weighted TI adjustment.

A valid multi-parameter model must therefore incorporate zonal renal architecture, applying distinct TPR-B/A covariance matrices to cortex, medulla, and pelvis rather than whole-organ averages that mask localized danger zones. Current edge devices lack calibrated B/A maps for renal tissue, although *ex vivo* measurements demonstrate that such mapping is technically feasible (34), and the low frame-rate requirements of renal perfusion imaging conflict with high-throughput DNN pruning originally optimized for cardiac or obstetric applications.

Such zonal calibration is mechanistically necessary because medullary perfusion is an order of magnitude below cortical flow, so cortex-averaged indices systematically underestimate medullary heating. Addressing these organ-specific computational barriers-including renal-specific thermal conductivity models and corticomedullary differentiation algorithms-is essential before the QCE-UT framework can be validated in renal cohorts. Quantitative DCE-CEUS time-intensity analysis offers a clinical route to cortical perfusion mapping, although depth-dependent attenuation correction remains unresolved (56).

Priority areas include conducting robust multicenter trials to validate efficacy in well-defined patient groups, establishing consensus on standardized measurement protocols for critical parameters, and elucidating dose-response relationships to inform personalized application. Harmonized reporting standards for safety endpoints would further enable meaningful cross-study comparison. Addressing these priorities will be crucial for translating promising computational frameworks into reliable, patient-centered clinical tools. Economic feasibility also deserves explicit appraisal: consumer-grade GPUs suffice for the emulated solvers, but probe-side edge modules add per-unit hardware cost and power draw that procurement committees must weigh against still-unquantified safety gains (21, 45).

## 6 Conclusion

Taken together, this narrative synthesis indicates that integrating quantum-inspired computational frameworks with real-time, tissue-specific corrections represents a conceptually promising but currently preliminary direction for advancing diagnostic ultrasound safety profiling. The evaluated literature suggests these integrated approaches may reduce adverse bioeffects and enhance imaging precision, primarily by dynamically adjusting for factors like tissue perfusion (1, 12, 13). Consequently, this moves beyond the static limitations of conventional Mechanical and Thermal Indices, offering a pathway toward more personalized safety monitoring (15, 16).

Nevertheless, the current evidence is tempered by several considerations. The observed benefits must be interpreted alongside substantial methodological variability, including non-standardized TPR measurement protocols, inconsistent blinding procedures, and a preponderance of short-term, single-center evaluations (12, 14, 16). These limitations preclude definitive efficacy claims and underscore that the framework remains in an early translational phase requiring rigorous multicenter validation before clinical deployment. Future work should prioritize standardized, multi-center studies to confirm efficacy and safety in well-defined patient populations, thereby bridging the gap between promising technological synergy and robust clinical application (11, 13, 21).
